# Supplementary figures and images for: CSF1R-dependent macrophages control postnatal somatic growth and organ maturation
Source: PLoS Genet. 2021 Jun 3;17(6):e1009605. doi: 10.1371/journal.pgen.1009605 (PMC8205168; doi:10.1371/journal.pgen.1009605)

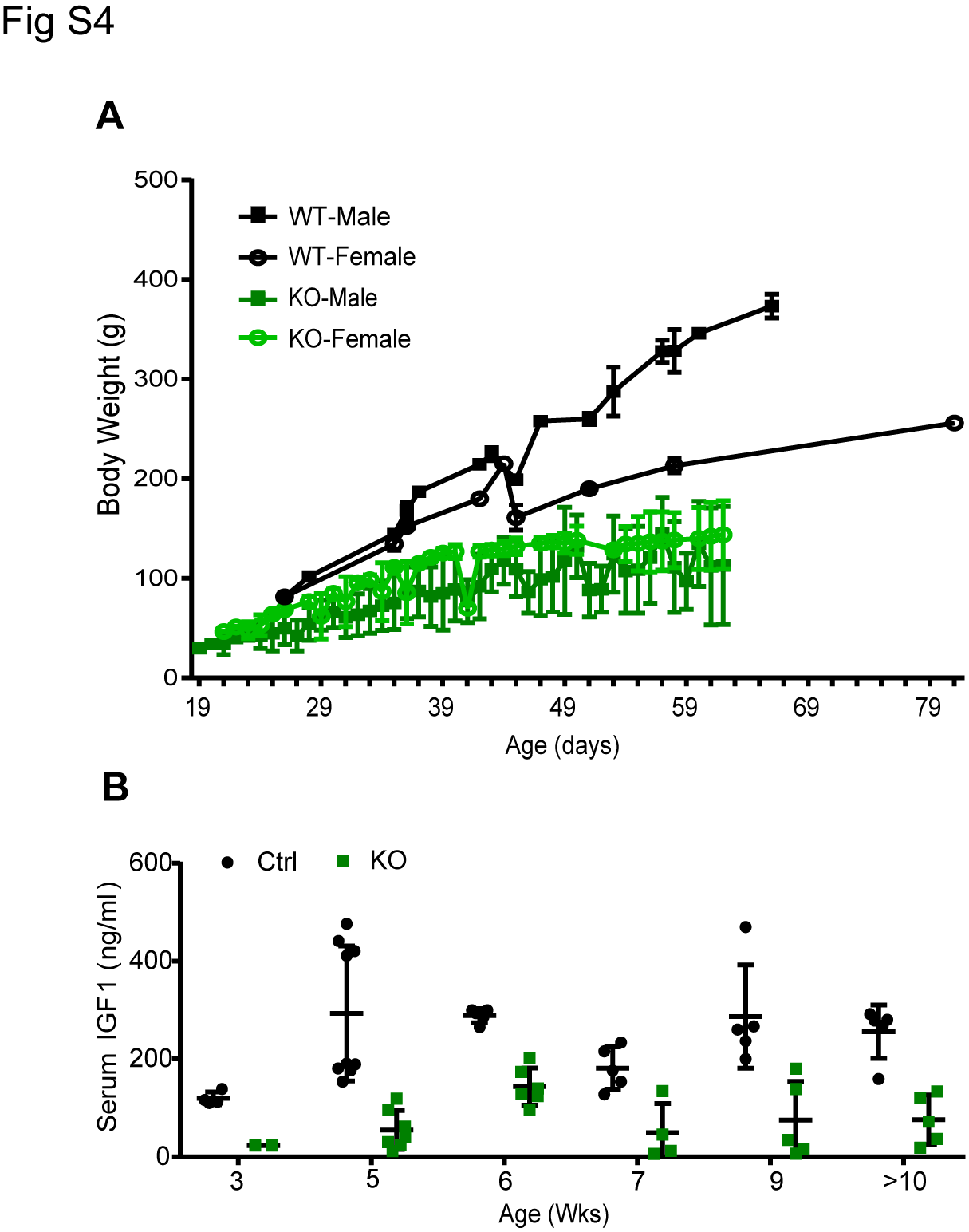

Supplement: S4 Fig — (A) Time course of postnatal body weight gain of outbred Sprague-Dawley (SD) male and female WT and Csf1rko rats. (B) Time course of serum IGF1 levels in a mixed cohort of male and female WT, Csf1rko SD rats. Serum was obtained at the ages indicated and IGF1 was measured by ELISA. (TIF) [file pgen.1009605.s004.tif]

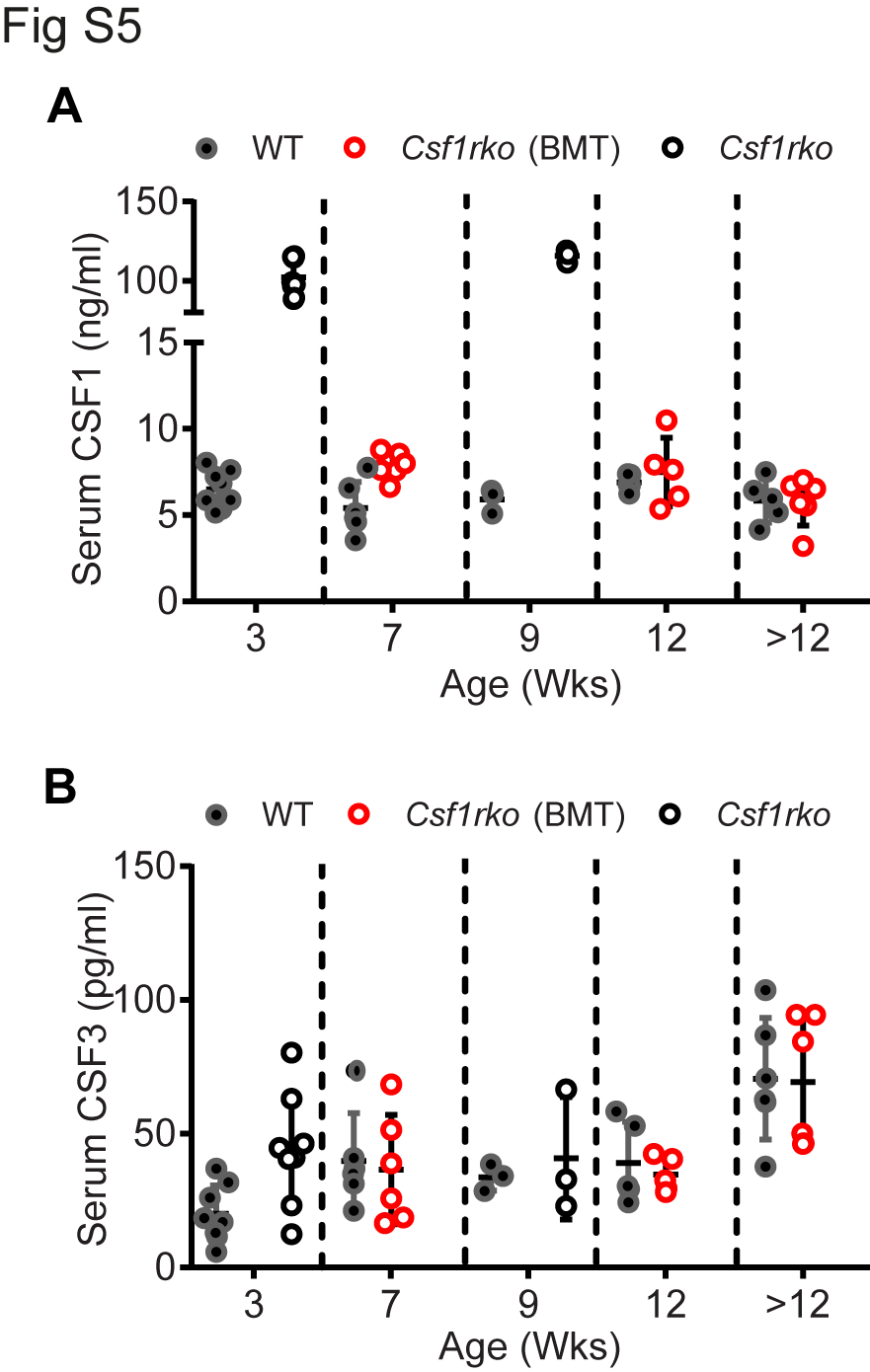

Supplement: S5 Fig — (A) Serum level of macrophage colony stimulating factor (CSF1) and (B) granulocyte colony stimulating factor (CSF3) in a mixed cohort of male and female WT, Csf1rko rats following BMT at 3 wks and Csf1rko rats at the ages indicated. (TIF) [file pgen.1009605.s005.tif]
